# miR-101a-3p/ROCK2 axis regulates neuronal injury in Parkinson’s disease models

**DOI:** 10.18632/aging.205836

**Published:** 2024-05-21

**Authors:** Xiang Tao, Wenfei Zhang, Chen Chen, Yang Tao, Yun Tao, Zhibiao Chen, Ge Zhang

**Affiliations:** 1 Department of Neurosurgery, Renmin Hospital of Wuhan University, Wuhan, Hubei 430060, China; 2 Department of Orthodontics, Wuhan First Stomatological Hospital, Wuhan, Hubei 430060, China; 3 Department of Nursing, Renmin Hospital of Wuhan University, Wuhan, Hubei 430060, China; 4 Department of Stomatology, Wuhan Central Hospital, Wuhan, Hubei 430060, China

**Keywords:** miR-101a-3p, ROCK2, Parkinson’s disease, α-synuclein

## Abstract

Background: Parkinson’s disease (PD) is a neurodegenerative disease characterized by the loss of dopaminergic neurons in substantia nigra pars compacta (SNpc). This study focuses on deciphering the role of microRNA (miR)-101a-3p in the neuronal injury of PD and its regulatory mechanism.

Methods: We constructed a mouse model of PD by intraperitoneal injection of 1-methyl 4-phenyl 1, 2, 3, 6-tetrahydropyridine hydrochloride (MPTP), and used 1-methyl-4-phenylpyridinium (MPP+) to treat Neuro-2a cells to construct an *in-vitro* PD model. Neurological dysfunction in mice was evaluated by swimming test and traction test. qRT-PCR was utilized to examine miR-101a-3p expression and ROCK2 expression in mouse brain tissues and Neuro-2a cells. Western blot was conducted to detect the expression of α-synuclein protein and ROCK2 in mouse brain tissues and Neuro-2a cells. The targeting relationship between miR-101a-3p and ROCK2 was determined by dual-luciferase reporter gene assay. The apoptosis of neuro-2a cells was assessed by flow cytometry.

Results: Low miR-101a-3p expression and high ROCK2 expression were found in the brain tissues of PD mice and MPP+-treated Neuro-2a cells; PD mice showed decreased neurological disorders, and apoptosis of Neuro-2a cells was increased after MPP+ treatment, both of which were accompanied by increased accumulation of α-synuclein protein. After miR-101a-3p was overexpressed, the neurological function of PD mice was improved, and the apoptosis of Neuro-2a cells induced by MPP+ was alleviated, and the accumulation of α-synuclein protein was reduced; ROCK2 overexpression counteracted the protective effect of miR-101a-3p. Additionally, ROCK2 was identified as the direct target of miR-101a-3p.

Conclusion: MiR-101a-3p can reduce neuronal apoptosis and neurological deficit in PD mice by inhibiting ROCK2 expression, suggesting that miR-101a-3p is a promising therapeutic target for PD.

## INTRODUCTION

Parkinson’s disease (PD) ranks second after Alzheimer’s disease (AD) as the commonest neurodegenerative disease, and it is characterized by the loss of dopaminergic neurons in substantia nigra pars compacta (SNpc) and the accumulation of α-synuclein in Lewy bodies [1, 2]. Currently, in clinical practice, levodopa remains the most effective drug for controlling the symptoms of PD; in addition, monoamine oxidase B/catechol-O-methyltransferase inhibitor is the alternative for treating PD, but these drugs cannot block the degeneration of dopaminergic neurons [3, 4]. In this context, the development of more effective drugs is still the primary goal of PD prevention and treatment.

As a kind of small non-coding RNA with a length of 20–22 nucleotides, microRNAs (miRNAs) participate in mRNA cleavage or post-transcriptional silencing via targeting messenger RNA (mRNA) 3′-untranslated region (3′UTR), thus regulating the expression of related genes [5, 6]. MiRNAs are dysregulated in many diseases including PD [7]. For instance, in PD patients, miR-155-5p expression is increased, while miR-146a-5p expression is decreased [8]. MiR-101a-3p is an important miRNA. Some studies have found that inhibiting miR-101a-3p can promote myocardial infarction [9], and miR-101a-3p is also reported to participate in neuron apoptosis after ischemic brain injury [10]. Notably, miR-101a-3p has been shown to delay the occurrence of AD by suppressing amyloid precursor protein (APP) expression [11], which suggests that miR-101a-3p exerts neuroprotective function in neurodegenerative diseases.

Rho-associated coiled-coil containing protein kinase 2 (ROCK2), a serine-threonine protein kinase, is a downstream effector of Rho GTPases, which can affect the contraction and movement of various cells in brain, including endothelial cells, vascular smooth muscle, neurons, neuroglia and so on. Besides, ROCK2 is regarded as the potential therapeutic target for various neurological diseases, such as traumatic brain injury [12], spinal cord injury [13], subarachnoid hemorrhage [14], and neurodegenerative diseases [15, 16]. The expression levels of ROCK1 and ROCK2 are increased in the brain tissue of dead PD patients [17–19]. So, inhibition of ROCK activity is considered as a potential strategy for the treatment of PD. It has been found that in the mouse PD model, oral administration of ROCK inhibitor fasudil activates Akt signaling pathway, reduces the death of dopaminergic neurons, and maintains the integrity of axons [20]. Additionally, in pre-clinical research, down-regulating ROCK2 delays PD progression [21, 22].

This study explored the possible mechanisms of miR-101a-3p and ROCK2 in PD pathogenesis to provide new targets for PD prevention and treatment.

## MATERIALS AND METHODS

### Animals

C57B/6J mice (8 weeks old; weight: 20–30 g) were obtained from the Model Animal Research of Wuhan University. The mice were kept in a 12-h light/dark cycle with food and water available ad libitum at a temperature of 22–25°C. All experiments were approved by the Animal Ethics Committee of Renmin Hospital of Wuhan University (approved on March 9th, 2023) and performed according to the Guide for the Care and Use of Laboratory Animals by the National Institutes of Health and the American Physiological Society.

### Mouse model of PD and micro-injection

The mouse model of PD was constructed as previously described [23, 24]. Mice were injected intraperitoneally with normal saline (control) or 1-methyl 4-phenyl 1, 2, 3, 6-tetrahydropyridine hydrochloride (MPTP, M325913; Toronto Research Chemicals, Canada) 4 times at intervals of 2 h on day 0. The MPTP dose for the mouse was 20 mg/kg each time. On the 3rd day, the mice were tested in the experiments. The intracerebroventricular injection of miR-101a-3p mimics was performed according to the previously described method [25]. Briefly, the mice were anesthetized and fixed. A small hole (2.5–3.0 mm deep) was made 0.5 mm behind the bregma and 1 mm laterally, and then a micro-syringe (Hamilton, NV, USA) was employed for intracerebroventricular injection of miR-101a-3p mimics (RiboBio, Guangzhou, China). MiR-101a-3p mimics (dissolved in 1.25 μL of Entranster™ *in vivo* transfection reagent (Engreen, Beijing, China)) were injected 48 h before MPTP treatment at a dose of 2.5 μg/2.5 μL per mouse. After the behavioral experiments, the mice were euthanized and beheaded, and then SNpc tissue was isolated from the brain of the mouse with microsurgical procedure and cryopreserved at −80°C.

### miRNA expression profile analysis

“miRNA Parkinson” was searched in GEO database (http://www.ncbi.nlm.nih.gov/geo/), and the dataset GSE16658 (which contains miRNA expression profile data of peripheral blood mononuclear cells from 19 Parkinson’s patients and 13 controls) was obtained. The data were analyzed according to the criterion “*P* < 0.05 and Log_2_(Fold Change) >1 or Log_2_(Fold Change) <−1” to screen out differentially expressed miRNAs.

### Cell culture, treatment and transfection

Mouse neuroblastoma cell Neuro-2a (ATCC, Manassas, VA, USA) was utilized to establish an *in-vitro* PD model. The cells were cultured in an incubator containing 5% CO_2_ at 37°C. The medium was Dulbecco’s modified Eagle’s medium (DMEM; HyClone, Logan, UT, USA) containing 10% fetal bovine serum (v/v) (Gibco, Grand Island, NY, USA) and 0.1% gentamycin (w/v) (HyClone, Logan, UT, USA). Neuro-2a cells were exposed to 1 mM 1-methyl-4-phenylpyridinium (MPP^+^) for 24 h to construct an *in-vitro* PD model [26, 27]. From GenePharma (Shanghai, China), the oligonucleotides and plasmids were obtained. Neuro-2a cells were transfected with ROCK2 plasmids employing Lipofectamine™ 3000 (Invitrogen, Carlsbad, CA, USA) 24 h before exposure to MPP^+^.

### Swimming test

The mouse was placed in a glass swimming tank (length: 40 cm; width: 25 cm; height: 16 cm), to conduct a swimming test. The tank was filled with water (temperature: 22–25°C) to a depth of 12 cm. The scoring rules are: 0, no swimming with head above the surface of water; 1, occasional swimming while floating with hind paws; 2, alternation between swimming float and passive floating; 3, swim continuously. The higher the score, the better the mouse’s condition [28].

### Traction test

The muscular strength and balance of mice were tested through traction test as previously described [29]. Specifically, 7 days after the PD model was constructed, the mouse’s front paws were put on a horizontal rope (diameter: 5 mm) about 70 cm from the ground. The scoring rules are as follows (according to the mouse’s hindlimbs grasping the rope): 0, fall from the rope; 1, no hind paw grasps the rope; 2, one hind paw grasps the rope; 3, both hindlimbs grasp the rope. The higher the score, the better the mouse’s muscular strength and balance.

### Rotarod test

The rotarod test was applied for measuring the motor coordination and balance of the mice [30]. The mouse was placed on a rod with a diameter of 3 cm, and the rotation speed of the rod was increased from 4 rpm to 40 rpm within 5 min. The mice were tested 2 times a day and 3 times per mouse at intervals of 15 min. Training experiments were performed 3 days before modeling. The motor function recovery test was performed 7 days after the PD model was constructed, and the time when the mice fell off was recorded. The longer the mouse stays, the better its motor coordination and balance abilities.

### Western blot assay

RIPA lysis buffer (Beyotime, Shanghai, China) was utilized to lyse SNpc tissues or Neuro-2a cells to extract total protein, and then a bicinchoninic acid protein assay kit (Beyotime, Shanghai, China) was employed to determine the protein concentration. The protein samples were mixed with the loading buffer, and heated in boiling water for protein denaturation. Next, sodium dodecyl sulfate polyacrylamide gel electrophoresis was performed, and subsequently the proteins were transferred to a polyvinylidene difluoride (PVDF) membrane (Millipore Corporation, Bedford, MA, USA). The PVDF membrane, after being blocked with 5% skim milk for 1 h at room temperature, was incubated overnight with the corresponding primary antibodies (α-Synuclein Rabbit mAb #4179, ROCK2 Rabbit mAb #9029, PTEN Rabbit mAb #9188, Akt Rabbit mAb #4691, Phospho-Akt Rabbit mAb #4060, GAPDH Rabbit mAb #5174, Cell Signaling Technology, MA, USA) at 4°C. After that, the membranes were incubated with the secondary antibody (anti-rabbit IgG, HRP-linked antibody #7074) for 1.5 h at room temperature. Eventually, the enhanced chemiluminescence kit (Beyotime, Shanghai, China) was used for developing the protein bands, and ImageJ software (NIH, Bethesda, MD, USA) was adopted to analyze the relative amount of protein, with GAPDH as the internal reference.

### Dual-luciferase reporter gene assay

The binding site of miR-101a-3p to ROCK2 3′UTR was predicted using TargetScan version 7.2 (http://www.targetscan.org/vert_72/). Wild type (WT) and mutant type (MUT) ROCK2 3′UTR were synthesized according to the binding sequence. Then they were inserted into the plasmid pGL3-control vector (Promega, Madison, WI, USA) to construct luciferase reporter gene plasmids (WT-ROCK2 and MUT-ROCK2). Subsequently, the plasmids were respectively co-transfected with miR-101a-3p mimic or control (miR-control) into 293T cells. Ultimately, the luciferase reporter gene detection system (E1910, Promega, Madison, WI, USA) was used for detection of the luciferase activity.

### Quantitative real-time PCR (qRT-PCR)

TRIzol reagent (Invitrogen, Carlsbad, CA, USA) was employed to extract total RNA from SNpc tissues and Neuro-2a cells. The RNA was reverse-transcribed into cDNA with a PrimeScript RT Reagent Kit (Perfect Real Time) (Takara, Otsu, Japan). Quantitative analysis of gene expression was carried out utilizing SYBR Premix Ex Taq™ II Kit (Takara, Otsu, Japan). The expression level of miR-101a-3p was normalized by U6, and the expression level of ROCK2 was normalized by GAPDH. The relative expression level was calculated by the 2^−ΔΔCt^ method. Below are the primer sequences (F: forward; R: reverse):

miR-101a-3p F: 5′-TGGGCTACAGTACTGTGATA-3′; miR-101a-3p R: 5′-TGCGTGTCGTGGAGTC-3′; ROCK2 F: 5′-TTGGTTCGTCATAAGGCATCAC-3′; ROCK2 R: 5′-TGTTGGCAAAGGCCATAATATCT-3′; U6 F: 5′-CGCTTCACGAATTTGCGTGTCAT-3′: U6 R: 5′-GCTTCGGCACATATACTAAAAT-3′; GAPDH F: 5′-GGGAAGCCCATCACCATCTTC-3′; GAPDH R: 5′-AGAGGGGCCATCCACAGTCT-3′.

### Cell apoptosis assay

Cell apoptosis was detected with the Annexin V-FITC/propidium iodide (PI) double staining apoptosis detection kit (Beyotime, Shanghai, China). After Neuro-2a cells were collected and washed with phosphate buffer saline, they (3 × 10^5^ cells in each sample) were resuspended, and 5 μL of Annexin V-FITC and 5 μL of PI were added in the suspension. The cell suspension was fully mixed, and incubated for staining at 4°C in the dark for 30 min. The stained cells were washed 3 times with binding buffer to remove excessive dye, and then resuspended in 500 μL of binding buffer. The samples were examined on BD FACS Canto II (BD Biosciences, San Jose, CA, USA) within 1 h, and the data were analyzed by FlowJo version 10.2 software (FlowJo, LLC Ltd., Ashland, OR, USA).

### Statistical analysis

GraphPad Prism 7.0 software (GraphPad Software Inc., La Jolla, CA, USA) was the data analysis tool, and the results were shown as “mean ± standard deviation (SD)”. Unpaired student’s *t*-test was conducted for comparison between two groups. For more than two groups, one-way ANOVA was performed, followed by Tukey’s post-hoc test. Statistical significance was defined as *P* < 0.05.

### Data availability statement

The data used to support the findings of this study are available from the corresponding author upon request.

## RESULTS

### miR-101a-3p is significantly down-regulated in PD

**Figure 1:**
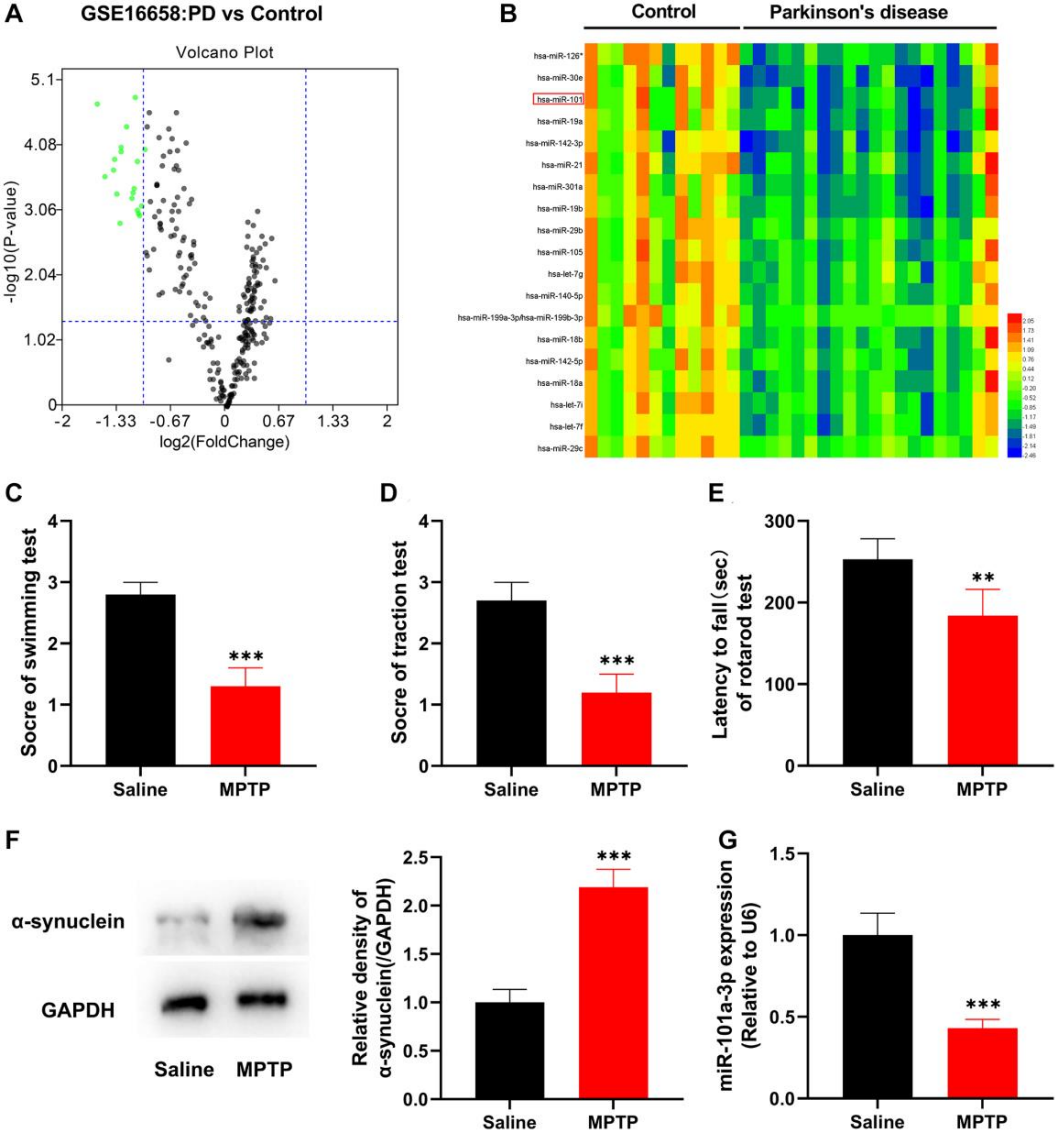
**miR-101a-3p is lowly expressed in peripheral blood mononuclear cells of PD patients and brain tissues of mice with PD.** (**A**) The volcano plot shows the differentially expressed miRNAs in the in peripheral blood mononuclear cells of PD patients (vs. healthy controls) in the GSE16658 dataset. (**B**) The heat map shows the expression profile of significantly down-regulated miRNAs in GASE16658. (**C**) Swimming test was adopted to score the motor ability of mice in normal saline group and MPTP group (*n* = 3 in both groups). (**D**) Traction test was adopted to score the balance ability of mice in normal saline group and MPTP group (*n* = 3 in both groups). (**E**) Rotarod test was adopted to evaluate the balance ability of mice in normal saline group and MPTP group (*n* = 3 in both groups). (**F**) Western blotting was applied to detect the expression of α-synuclein protein in mice of the normal saline group and MPTP group (*n* = 3 in both groups). (**G**) qRT-PCR was applied to detect miR-101a-3p expression in SNpc of mice in the normal saline group and MPTP group (*n* = 3 in both groups). ^**^P < 0.01 and ^***^P < 0.001.

To study the role of miRNAs in PD, first of all, GSE16658 microarray dataset was analyzed through GEO2R, and it was revealed that, 19 miRNAs were significantly down-regulated in peripheral blood mononuclear cells of PD patients (Figure 1A). Among them, has-miR-101 was markedly down-regulated (Figure 1B), and has-miR-101 corresponds to mmu-miR-101a-3p (miR-101a-3p) in mice, so it was selected as the follow-up research target. Subsequently, we constructed a mouse model of PD by intraperitoneal injection of MPTP. Swimming test, traction test and rotarod test showed that as against the normal saline group, the scores of the mice in MPTP group were decreased remarkably, suggesting that PD mice had a significant decline in motor and balance, and showed severe neurological injury (Figure 1C–1E). Western blot indicated that compared with the normal saline group, the expression of the PD marker protein α-synuclein in the mice of the MPTP group was observably elevated (Figure 1F), which indicated that the PD mouse model was successfully established. qRT-PCR revealed that miR-101a-3p was markedly under-expressed in the SNpc of the mice in MPTP group compared with the normal saline group (Figure 1G). Collectively, the above data suggest that dysregulation of miR-101a-3p contributes to PD pathogenesis.

### miR-101a-3p inhibits ROCK2 expression

**Figure 2:**
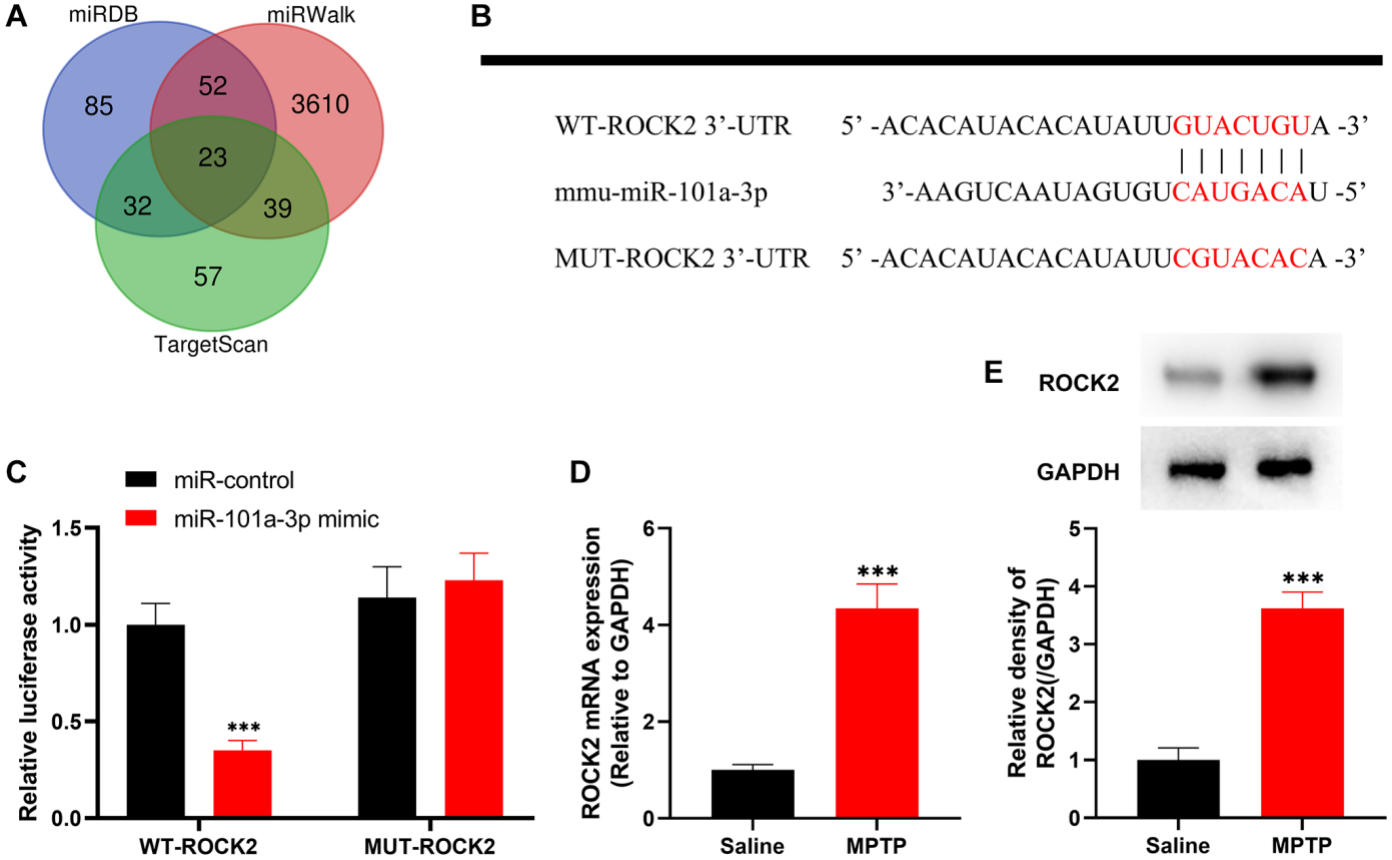
**ROCK2 is the target of miR-101a-3p.** (**A**) The Venn diagram shows the results of the miRDB, miRWalk and TargetScan databases to predict possible downstream targets of mmu-miR-101a-3p. (**B**) Bioinformatics analysis was conducted to predict the binding site between miR-101a-3p and ROCK2, and WT-ROCK2 and MUT-ROCK2 luciferase reporter gene vectors were constructed. (**C**) 293T cells were co-transfected with miR-101a-3p mimic or miR-control and WT-ROCK2 or MUT-ROCK2. After 48 h, the luciferase activity of each group of cells was determined. (**D**) ROCK2 mRNA expression levels of the mice in the normal saline group and MPTP group were detected by qRT-PCR. (**E**) Western blot was performed to detect ROCK2 protein expression level in the normal saline group and MPTP group. ^***^P < 0.001.

Next, we explored miR-101a-3p’s downstream targets. miRDB, miRWalk and TargetScan databases were searched to predict the possible downstream targets of miR-101a-3p, among which ROCK2 is reported to be associated with the pathogenesis of nervous system diseases [12–19] (Figure 2A). The binding site of miR-101a-3p with ROCK2 3′UTR was predicted through the TargetScan database, and accordingly, the luciferase reporter gene vectors were constructed (Figure 2B). Subsequently, the luciferase reporter gene assay indicated that compared with the miR-control group, miR-101a-3p mimics markedly reduced the luciferase activity of the cells transfected with WT ROCK2 reporter, yet failed to significantly affect that of MUT ROCK2 reporter (Figure 2C). Then, ROCK2 expression in the SNpc of the mice in two groups was detected by qRT-PCR and Western blot assays, and it was revealed that compared with the normal saline group, ROCK2 expression in MPTP group was markedly increased (Figure 2D, 2E). The above-mentioned evidence suggests that miR-101a-3p suppresses ROCK2 expression, and their dysfunction may contribute to PD pathogenesis.

### miR-101a-3p mimic represses ROCK2 expression and alleviates neurological damage of PD mice

**Figure 3:**
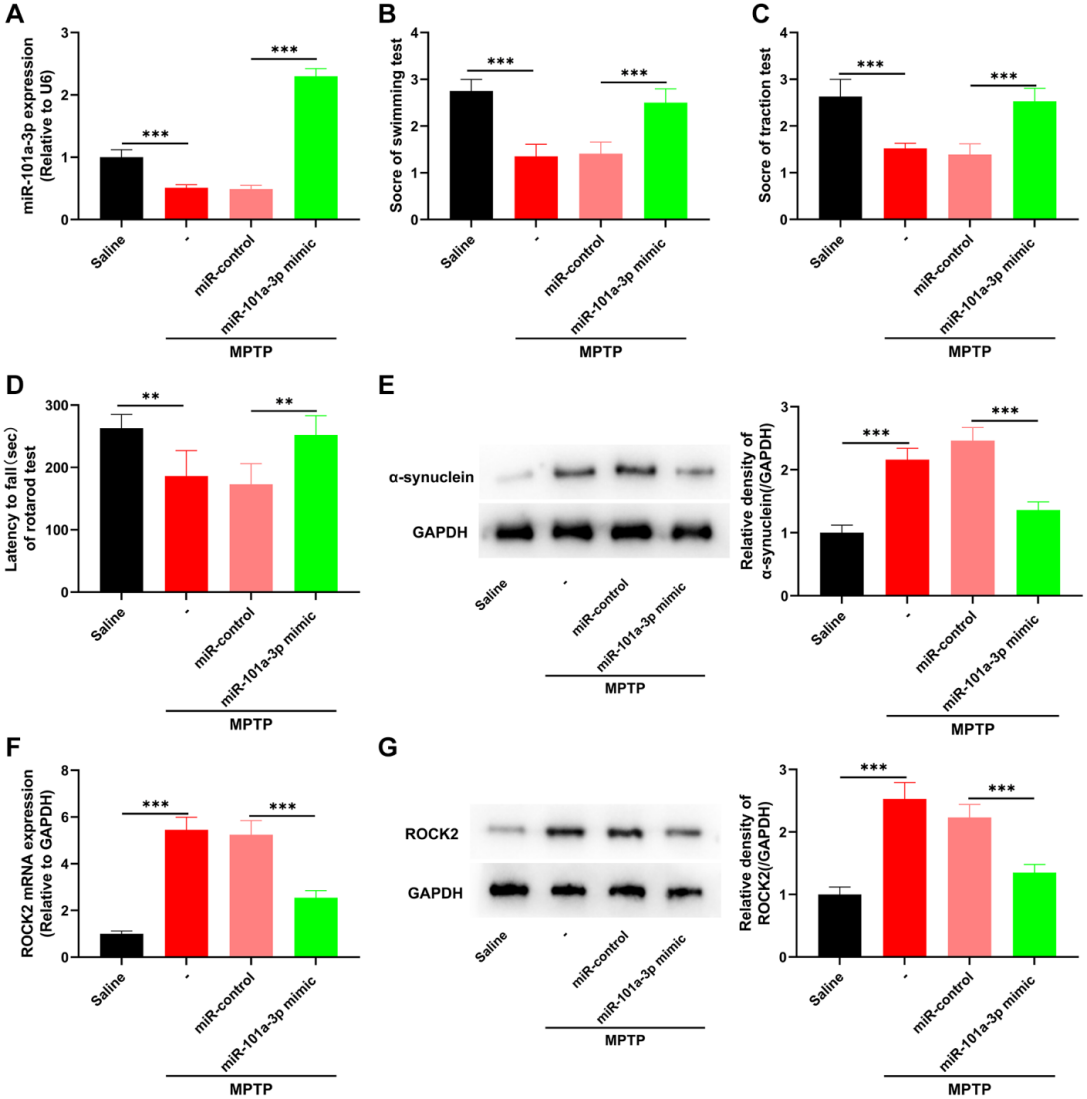
**miR-101a-3p mimic inhibits ROCK2 expression and neurological damage in PD mice.** To confirm the role of miR-101a-3p in PD mice, miR-101a-3p mimics were intracerebroventricularly injected into the mice to overexpress miR-101a-3p before intraperitoneal injection of MPTP into the mice. There were 4 groups: saline group, MPTP group, MPTP+miR-control group, and MPTP+miR-101a-3p mimic group (*n* = 3 in each group). (**A**) qRT-PCR was performed to detect the expression levels of miR-101a-3p in mice in each group. (**B**) Swimming test was conducted to score the motor ability of each group of mice. (**C**) Traction test was conducted to score the balance ability of each group of mice. (**D**) Rotarod test was conducted to score the balance ability of each group of mice. (**E**) Western blot was performed to detect α-synuclein protein expression in each group of mice. (**F**) Detection via qRT-PCR of ROCK2 mRNA expression level in each group of mice. (**G**) Western blot detection of ROCK2 protein expression levels in each group of mice. ^**^P < 0.01 and ^***^P < 0.001.

To confirm the role of miR-101a-3p in PD, we overexpressed miR-101a-3p in mouse brain tissues by intracerebroventricular injection of miR-101a-3p mimics, and then evaluated the neurological function of mice. In comparison to MPTP+miR-control group, miR-101a-3p was remarkably up-regulated in SNpc tissues of mouse after miR-101a-3p overexpression (Figure 3A). Swimming test, traction test and rotarod test showed that in comparison with MPTP+miR-control group, the scores of mice after miR-101a-3p overexpression were increased significantly (Figure 3B–3D). Furthermore, compared with the MPTP+miR-control group, the expression of PD marker protein α-synuclein in mice overexpressing miR-101a-3p was markedly reduced (Figure 3E). qRT-PCR and Western blot showed that compared with the MPTP+miR-control group, ROCK2 expression in mice overexpressing miR-101a-3p was decreased significantly (Figure 3F, 3G). The aforementioned findings imply that miR-101a-3p mimic represses ROCK2 expression in PD mice and mitigates the neurological injury of PD mice.

### Dysregulation of miR-101a-3p and ROCK2 in neruo-2a cells treated with MMP^+^

**Figure 4:**
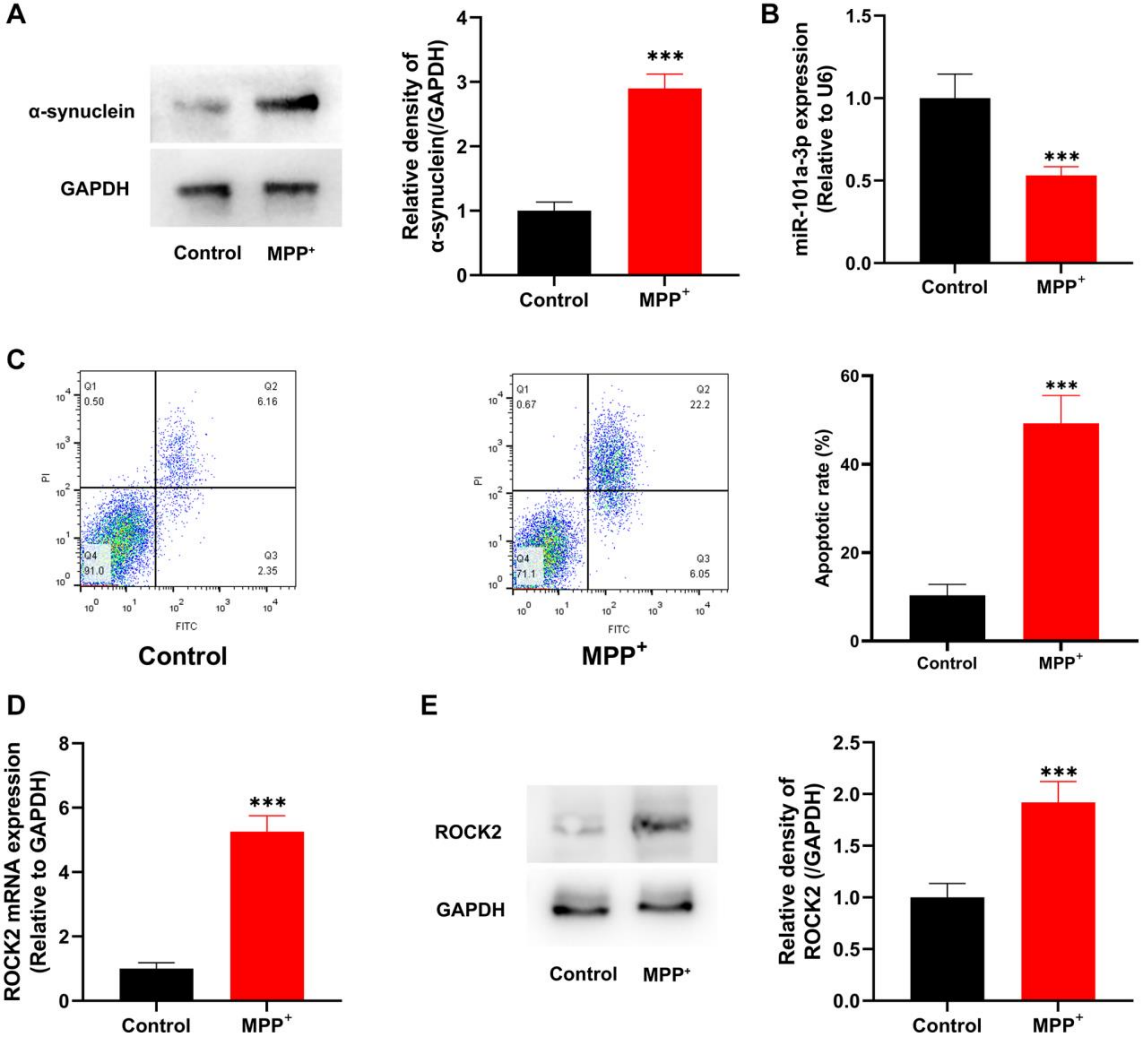
**MPP^+^ suppresses miR-101a-3p expression and induces the apoptosis of Neuro-2a cells.** (**A**) Western blotting was utilized to detect α-synuclein protein expression in control group and MPP^+^ group. (**B**) Detection of miR-101a-3p expression in Neuro-2a cells of the control group and MPP^+^ group by qRT-PCR. (**C**) Flow cytometry was utilized to detect the apoptosis level in control group and MPP^+^ group. (**D**) Detection of ROCK2 mRNA expression level in Neuro-2a cells in the control group and MPP^+^ group by qRT-PCR. (**E**) Western blot detection of ROCK2 protein expression level in Neuro-2a cells of control group and MPP^+^ group. ^***^P < 0.001.

Subsequently, Neuro-2a cells were treated with neurotoxin MPP^+^ to construct an *in vitro* model of PD. Western blot showed that compared with the control group, the expression of α-synuclein protein in the cells of MPP^+^ group was remarkably enhanced (Figure 4A). qRT-PCR revealed that as against the control group, miR-101a-3p expression in MPP^+^ group was observably lowered (Figure 4B). Flow cytometry was performed to detect cell apoptosis, and in contrast to the control group, the apoptosis level of the cells in MPP^+^ group was noticeably elevated (Figure 4C). qRT-PCR and Western blotting showed that the ROCK2 mRNA and protein expression levels in MPP^+^ group were significantly higher compared with the control group (Figure 4D, 4E).

### miR-101a-3p mimic suppresses MPP^+^-induced apoptosis of Neuro-2a cells

**Figure 5:**
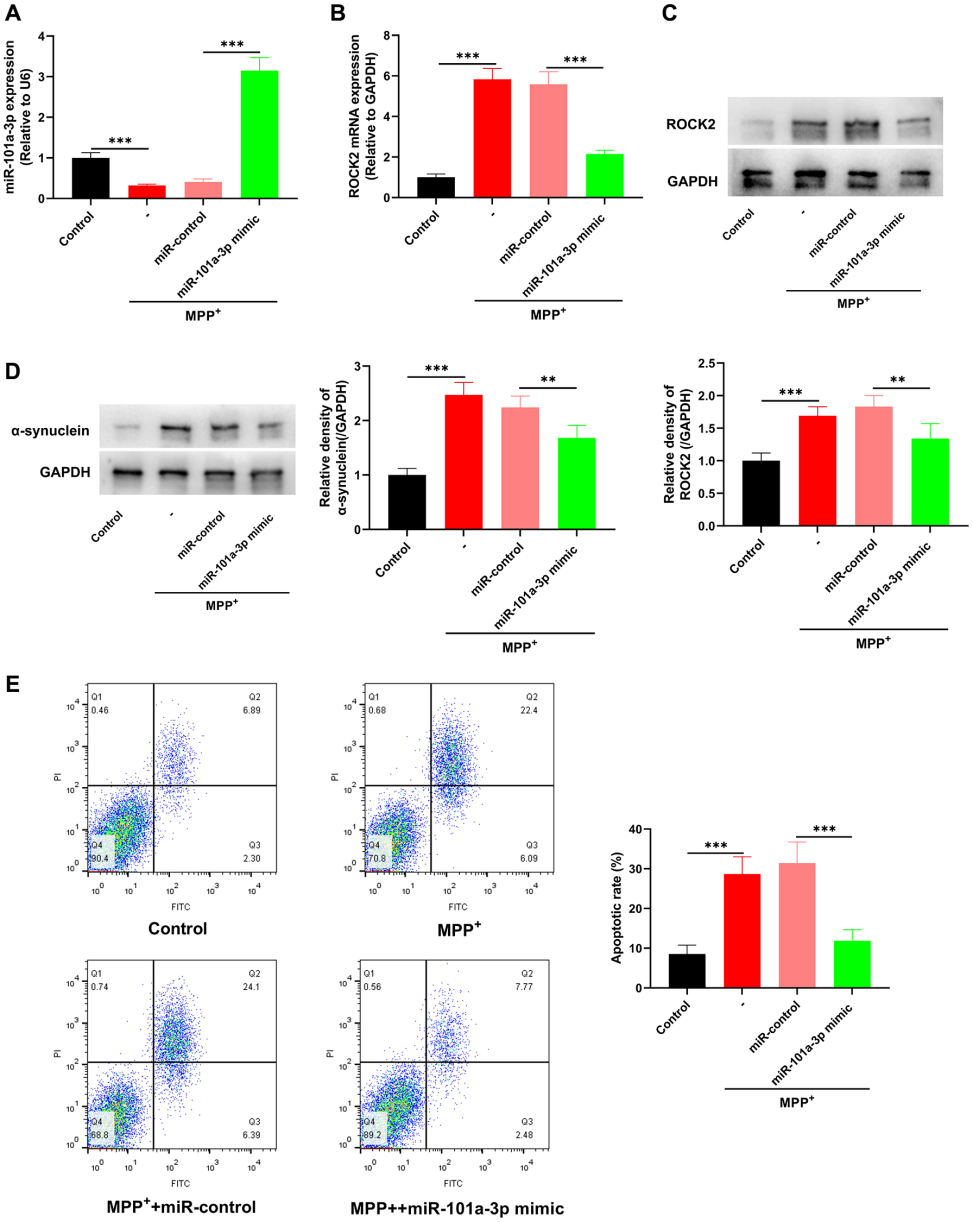
**miR-101a-3p mimic inhibits the apoptosis of Neuro-2a cells induced by MPP^+^.** To confirm the role of miR-101a-3p in Neuro-2a cells treated with MPP^+^, miR-101a-3p was overexpressed in Neuro-2a cells by transfecting miR-101a-3p mimics before Neuro-2a cells were exposed to MPP^+^. There were 4 groups: control group, MPTP group, MPP^+^+miR-control group, and MPP^+^+miR-101a-3p mimic group. (**A**) Detection via qRT-PCR of miR-101a-3p expression in each group of cells. (**B**) Detection of ROCK2 mRNA expression in each group of cells via qRT-PCR. (**C**) Western blot detection of ROCK2 protein expression level in each group of cells. (**D**) Western blot was utilized to detect α-synuclein protein expression in each group of cells. (**E**) Flow cytometry was utilized to detect the apoptosis level of Neuro-2a cells in each group. ^**^P < 0.01 and ^***^P < 0.001.

To determine the biological functions of miR-101a-3p in MPP^+^-induced Neuro-2a cells, Neuro-2a cells were transfected with miR-101a-3p mimics or miR-control before MPP^+^ treatment. qRT-PCR indicated that miR-101a-3p expression was markedly up-regulated in MPP^+^+miR-101a-3p group as against that in MPP^+^+miR-control group (Figure 5A), whereas ROCK2 expression in MPP^+^+miR-101a-3p group was markedly inhibited (Figure 5B, 5C). Moreover, compared with MPP^+^+miR-control group, the expression of PD marker α-synuclein in the MPP^+^+miR-101a-3p group was remarkably down-regulated (Figure 5D), and the apoptosis level of Neuro-2a cells was remarkably reduced after transfection of miR-101a-3p (Figure 5E). These findings suggest that miR-101a-3p mimic significantly inhibits ROCK2 expression in Neuro-2a cells and attenuates the apoptosis of Neuro-2a cells induced by MPP^+^.

### miR-101a-3p alleviates the injury of Neuro-2a cells treated with MPP^+^ by inhibiting ROCK2

**Figure 6:**
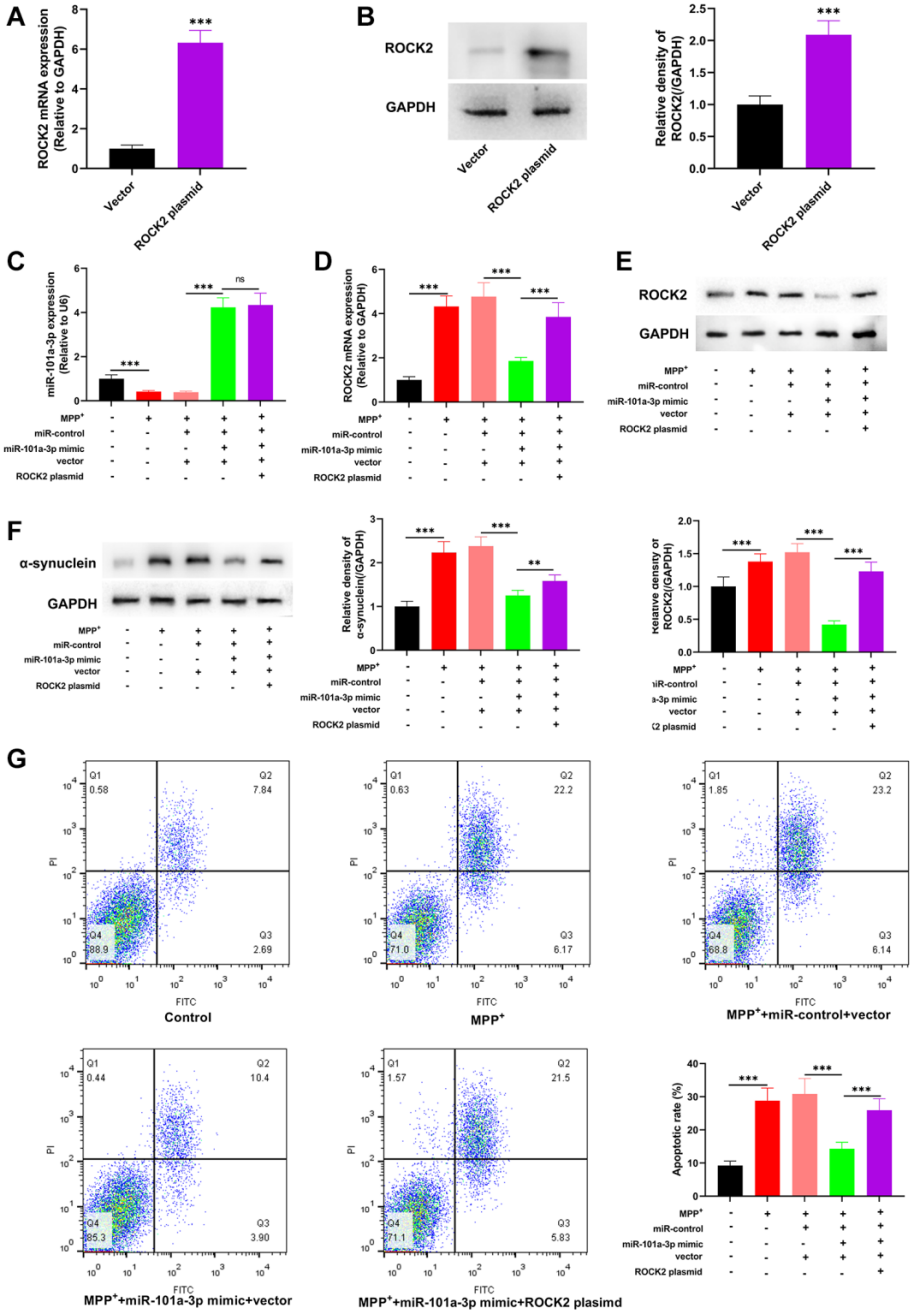
**miR-101a-3p reduces the damage of Neuro-2a cells induced by MPP^+^ via inhibiting ROCK2.** To confirm the mechanisms of miR-101a-3p and ROCK2 in MPP^+^-induced injury of Neuro-2a cells, miR-101a-3p mimics and ROCK2 overexpression plasmids were transfected into Neuro-2a cells before Neuro-2a cells were exposed to MPP^+^. There were 5 groups: control group, MPTP group, MPP^+^+miR-control+vector group, MPP^+^+miR-101a-3p mimic+vector group, and MPP^+^+miR-101a-3p mimic+ROCK2 plasmid group. (**A**) qRT-PCR was employed to verify the change in ROCK2 mRNA expression after Neuro-2a cells were transfected with ROCK2 overexpression plasmids. (**B**) Western blot was employed to examine the change in ROCK2 protein expression after Neuro-2a cells were transfected with ROCK2 overexpression plasmids. (**C**) Detection of miR-101a-3p expression in each group of cells through qRT-PCR. (**D**) ROCK2 mRNA expression in each group of cells was detected by qRT-PCR. (**E**) Western blot detection of ROCK2 protein expression level in each group of cells. (**F**) Western blotting was conducted to detect α-synuclein protein expression in each group of cells. (**G**) Flow cytometry was conducted to detect the apoptosis level in each group. ^**^P < 0.01 and ^***^P < 0.001.

To decipher the role of miR-101a-3p/ROCK2 axis in mitigating PD-related neurological injury, we transfected ROCK2 overexpression plasmids into Neuro-2a cells, and after transfection, ROCK2 was highly expressed in Neuro-2a cells (Figure 6A, 6B). We then transfected miR-101a-3p mimics and miR-101a-3p mimics + ROCK2 overexpression plasmids into Neuro-2a cells, respectively, and qRT-PCR confirmed that in comparison to the MPP^+^+miR-101a-3p mimic+vector group, there was no significant change in miR-101a-3p expression in the MPP^+^+miR-101a-3p mimic+ROCK2 plasmid group (Figure 6C). qRT-PCR and Western blotting indicated that in comparison with the MPP^+^+miR-101a-3p mimic+vector group, ROCK2 expression in the MPP^+^+miR-101a-3p mimic+ROCK2 plasmid group was increased remarkably (Figure 6D, 6E), and α-synuclein protein expression was also increased significantly (Figure 6F). Moreover, flow cytometry showed that compared with MPP^+^+miR-101a-3p mimic+vector group, the apoptosis level of the cells in MPP^+^+miR-101a-3p mimic+ROCK2 plasmid group was markedly increased (Figure 6G). The above-mentioned data suggest that miR-101a-3p can relieve MPP^+^-intoxicated Neuro-2a cell damage via inhibiting ROCK2 expression.

## DISCUSSION

1–2‰ of the population is affected by PD; the morbidity of PD rises with age [31]. Based on aging alone, as many as 700,000 PD cases are predicted by 2040 [32]. Increasing studies suggest that dysregulation of certain specific miRNAs may be related to the pathogenesis of neurodegenerative diseases such as PD [33]. For instance, miR-155 acts as a treatment target to regulate α-synuclein-induced inflammatory reaction in the PD model [34]. MiR-7 modulates Nod-like receptor protein 3 (NLRP3)-mediated neuroinflammation in the development of PD [35]. MiR-22 plays a neuroprotective role in the 6-hydroxydopamine (6-OHDA)-induced PD cell model via targeting transient receptor potential melastatin 7 (TRPM7) [36]. Loss of miR-425 facilitates necroptosis and dopaminergic neurodegeneration in PD [30]. Bioinformatics analysis in the present work suggested that, besides miR-101a-3p, multiple miRNAs were also dysregulated in peripheral blood mononuclear cells of PD patients, including but not limited to miR-126, miR-30e, miR-19a, miR-142-3p, miR-21, miR-301a, miR-19b, miR-29b, miR-105, let-7g and miR-140-5p, some of which have been reported to regulate PD pathogenesis and progression via different mechanisms [37–41]. Previous studies have reported that miR-101a-3p partakes in multiple neurological diseases [10, 11, 42, 43]. This study confirmed that miR-101a-3p is lowly expressed in the MPTP-induced mouse model of PD and Neuro-2a cells exposed to MPP^+^. We further confirmed that miR-101a-3p mimics can alleviate MPTP-induced damage to the neurological behaviors of the mice, and ameliorate MPP^+^ exposure-induced neuronal apoptosis, indicating that it has a neuroprotective effect in PD. In the present work, miR-101a/miR-101a-3p was found to be down-regulated in cell model of PD, animal model of PD and peripheral blood mononuclear cells of PD patients. However, whether miR-101a is dysregulated in the SNpc of PD patients, is not studied, due to the lack of clinical samples. Notably, a recent study reports that miR-101a-3p overexpression impairs synaptic plasticity and contributes to synucleinopathy [44], suggesting miR-101a-3p may exert different biological functions in different biological processes during PD pathogenesis. In the future, the expression pattern of miR-101a in the brain of the patients deserves investigation. Additionally, different gene-editing animal models may be helpful to further clarify the role of miR-101a-3p in PD progression.

This study confirmed that ROCK2 was miR-101a-3p’s downstream target and its expression was up-regulated in PD models. ROCK1 and ROCK2 are isoforms as the downstream effectors of the small GTP-binding protein Rho; ROCK1 is mainly found in the lungs, liver, testis, blood and immune system, while ROCK2 is mainly expressed in brain and muscle [45]. ROCK2 is a crucial player in neuronal death and axon regeneration, and is believed to play a key part in PD development [46, 47]. Therefore, multiple ROCK2 inhibitors are considered promising targets for neuroprotective therapy against PD [48–50]. As non-coding RNA research evolves in recent years, miRNAs have been proved to induce mRNA degradation or repress translation by binding to the 3′UTR of the target mRNA, so as to modulate the expression of downstream target genes and participate in modulating PD development. For instance, miR-291 plays a protective role in neuron degeneration through modulating ROCK2 expression [51]. MiR-135a-5p/ROCK2 axis may play a role in the protective effect of hydrogen sulfide against PD [52]. The present study confirmed that miR-101a-3p reduces neuronal apoptosis and neurological deficits in PD mice by regulating ROCK2 expression. Of course, a single miRNA targets multiple downstream genes, and beside ROCK2, the other downstream targets of miR-101a-3p in PD pathogenesis awaits to be explored in the future. Additionally, the downstream mechanism of miR-101-3p/ROCK2 in modulating PD pathogenesis is still obscure. Interestingly, some recent studies have found that there is a mutual regulatory relationship between ROCK2 and PINK1/Parkin axis. In a Drosophila PD model, ROCK2 inhibitor or ROCK2 knockout promotes Parkin recruitment from cytoplasm to mitochondria, enhances mitochondrial autophagy and reduces nerve damage, and finally enhances the climbing ability of drosophila and alleviates PD symptoms [53]. Another study reports that silencing PINK1 expression in hippocampal neurons induces increased ROCK2 expression, accompanied by abnormal morphology of dendritic spines [54]. These studies imply that miR-101a-3p/ROCK2 axis may interact with PINK1/Parkin axis to regulate PD pathogenesis, which deserves further investigation in the following work.

## CONCLUSION

To sum up, miR-101a-3p suppresses neuronal apoptosis and reduces neurological dysfunction of PD via targeting ROCK2, by which it plays a neuroprotective role, implying that miR-101a-3p may act as a promising target for PD treatment.
